# Measures of satisfaction with care during labour and birth: a comparative review

**DOI:** 10.1186/1471-2393-13-108

**Published:** 2013-05-08

**Authors:** Alexandra Sawyer, Susan Ayers, Jane Abbott, Gillian Gyte, Heike Rabe, Lelia Duley

**Affiliations:** 1 School of Psychology, University of Sussex, Brighton, East Sussex, UK; 2 School of Health Sciences, City University London, 20 Bartholomew Close, London, UK; 3 Jane Abbott, Bliss (The Special Care Baby Charity), 9 Holyrood Street, London, UK; 4 Gillian Gyte, National Childbirth Trust, Alexandra House, Oldham Terrace, London, UK; 5 Heike Rabe, Academic Department of Paediatrics, Brighton and Sussex University Hospitals Trust, Royal Alexandra Children’s Hospital, Eastern Road, Brighton, UK; 6 Lelia Duley, Nottingham Clinical Trials Unit, University of Nottingham, Nottingham, UK

**Keywords:** Patient satisfaction, Labour, Birth, Questionnaire, Measurement

## Abstract

**Background:**

Satisfaction is the one of the most frequently reported outcome measures for quality of care. Assessment of satisfaction with maternity services is crucial, and psychometrically sound measures are needed if this is to inform health practices. This paper comparatively reviews current measures of satisfaction with care during labour and birth.

**Methods:**

A review of the literature was conducted. Studies were located through computerised databases and hand searching references of identified articles and reviews. Inclusion criteria were that the questionnaire was a multi-item scale of satisfaction with care during labour and birth, and some form of psychometric information (either information about questionnaire construction, or reliability, or validity) had to be reported.

**Results:**

Nine questionnaires of satisfaction with care during labour and birth were identified. Instruments varied in psychometric properties and dimensions. Most described questionnaire construction and tested some form of reliability and validity. Measures were generally not based on the main theoretical models of satisfaction and varied in scope and application to different types of samples (e.g. satisfaction following caesarean section). For an in-depth measure of satisfaction with intrapartum care, the Intrapartal-Specific Quality from the Patient’s Perspective questionnaire (QPP-I) is recommended. Brief measures with good reliability and validity are provided by the Six Simple Questions (SSQ) or Perceptions of Care Adjective Checklist (PCACL-R).

**Conclusions:**

Despite the interest in measures of satisfaction there are only a small number of validated measures of satisfaction with care during labour and birth. It is important that brief, reliable and valid measures are available for use in general and specific populations in order to assist research and inform practice.

## Background

Satisfaction is the of the most frequently reported outcome measures for quality of care [1] and enhanced satisfaction has been identified as a goal for improvement in health care. [2] Women’s satisfaction with maternity services, especially care during labour and birth, has become increasingly important to healthcare providers, administrators, and policy makers [3,4]. Research shows that women’s satisfaction with childbirth is partly related to the health and well-being of the mother and her baby. For example, dissatisfaction is associated with poorer postnatal psychological adjustment, a higher rate of future abortions, preference for a caesarean section, more negative feelings towards the infant and breast-feeding problems [1,5,6].

However, the concept of satisfaction is complex and poorly defined [7]. A definition suggested by Ware et al. [8] is that an individual’s satisfaction with healthcare is a “personal evaluation of healthcare services and providers” (p.247). These evaluations reflect the personal preferences of the individual, the individual’s expectations, and the realities of the care received. Linder-Pelz and Struening [9] provide a similar definition noting that satisfaction comprises of “multiple evaluations of distinct aspects of healthcare which are determined (in some way) by the individual’s perceptions, attitudes and comparison processes” (p. 42). This definition highlights the multidimensional nature of satisfaction.

Several theories of people’s satisfaction with healthcare have been developed [10]. The majority of studies on people’s satisfaction are based on fulfilment or discrepancy theories [3,11]. Fulfilment theories state that a person’s satisfaction is determined by the outcome of the experience, and previous expectations are not important. In comparison, discrepancy theories argue that a person’s satisfaction is determined by the differences between what is expected and what actually happens. Theories of people’s satisfaction can be used to inform the development of measures of satisfaction. However, the extent to which this is the case for measures of satisfaction with labour and birth is not clear.

The importance of assessing satisfaction when evaluating healthcare services means it is also imperative that reliable and valid measures are used [4]. Surveys are the most common method of assessing individual’s experiences of care in research, evaluation, and audits. Although satisfaction surveys are vital tools for accessing a person’s views, and can form an integral part of assessing the quality of care and informing service planning, they have not always been conducted with the necessary methodological rigour [4]. Firstly, many surveys only use a single item to assess satisfaction with care, which ignores the multidimensional quality of satisfaction [11,12]. For example, research looking at satisfaction with maternity care suggests the following dimensions are important: staff-woman interaction, information, involvement in decision making, pain relief, and birth environment [3,13-15]. Secondly, surveys of satisfaction with maternity services have been criticised for not being developed on the basis of theory [16]. Sitzia and Wood [7] argue that conceptual and theoretical issues should underpin the design and structure of a methodology. Thirdly, satisfaction measures in general have been criticised for being poorly constructed along with having poor psychometric properties including reliability and validity [17,18].

It is therefore evident that psychometrically sound measures are needed to appropriately evaluate satisfaction with care during labour and birth. However, available measures of satisfaction with childbirth are diverse and range from single item measures to extensive surveys of all aspects of maternity care. Measures do not always differentiate between the experience of labour and birth (such as pain and negative emotions) and the experience of care [17,19]. Therefore many research studies have created their own scales, with little or no psychometric evaluation. This has resulted in a confusing array of available measures that vary in content and quality. There is currently no review of questionnaires used to measure satisfaction with care during labour and birth. This paper therefore reviews current published measures of satisfaction with care during labour and birth. More specifically this review aimed to identify instruments which measure satisfaction with labour and birth; and to evaluate the psychometric properties of these questionnaires.

## Methods

### Criteria for selecting potentially eligible studies

Studies were included if they reported use of a questionnaire that was a multi-item scale of satisfaction with care during labour and birth, and provided psychometric information (either about questionnaire construction, or reliability, or validity) for the satisfaction measure. Studies were excluded if they reported questionnaires that: (1) described an omnibus measure to assess satisfaction with maternity services overall (e.g. antenatal, birth, and postnatal); (2) included items that were not specific to labour/birth; (3) were qualitative assessments of birth satisfaction; (4) were developed specifically for fathers; (5) comprised of dissertations, non-original research (i.e. reviews, opinion papers), or conference presentations; and (6) were not written in English.

### Search strategy

A systematic search was conducted to identify potentially eligible studies using the search terms: (Birth or Childbirth or Lab*r or Intrapart*) AND (Satisfaction or Perception or Evaluation) AND (Questionnaire or Measure* or Scale or Instrument). The databases Scopus, PsychArticles, PsychInfo, PubMed, and Web of Science were searched up to 30 July 2011. Reference lists in reports of included studies were searched for additional studies. Citations identified in this search strategy were then checked electronically to identify and remove duplicates. Finally, the Web of Knowledge and Scopus were searched for all reports that cited the final questionnaire measures. No new citations were identified.

### Selection of studies and data extraction

Titles and abstracts (where available) for each citation were screened by one review author (AS) and those clearly not eligible were excluded. Full text reports were retrieved for the remaining citations. These were screened for inclusion by independently by two review authors (AS and SA). For excluded studies, reasons for exclusion were recorded. For included studies, data were extracted onto a prepared data extraction form by AS, and checked by SA. Data extraction included: the satisfaction measure used, format of the questionnaire, country where study was conducted, sample, and questionnaire construction, reliability and validity.

### Assessment of psychometric quality

Psychometric quality of each questionnaire was assessed using the following criteria:

Questionnaire construction

• *Item generation* – this phase is undertaken to develop a pool of items that should include all important elements of satisfaction by reviewing existing questionnaires, literature, opinions from maternity care-providers and focus groups of mothers. Items taken directly from women represent what they truly value and opinions from providers can ensure that significant elements of care have not been missed [20].

• *Pilot study -* the process of pre-test and pilot testing of the final questionnaire by using the response from the pre-test group to make revisions to the final version. Items with ambiguous meanings can be eliminated to maximise the reliability and validity of the questionnaire. The questionnaire should then be re-administered and tested in a new sample [20].

Reliability – the ability of a measure to produce consistent results

• *Internal consistency –* refers to the extent to which items in a questionnaire are measuring the same things. One method of assessing internal consistency is using Cronbach’s alpha (α) and a minimum value of 0.70 is considered reliable [21].

• *Test–retest reliability* – refers to the ability of a test to yield consistent scores over time. It is recommended that a minimum value of 0.70 is considered reliable [22].

Validity –the extent a questionnaire measures what it is supposed to measure

• *Content validity –* whether an instrument adequately covers the domains to be evaluated. The development of a content valid instrument is typically achieved by analysis of the instrument by raters familiar with the construct of interest. Content validity can also be assessed by focus group participants and review of the literature. Another source of evidence can be obtained from how the measure was initially developed.

• *Face validity –* is closely related to content validity and refers to whether a measure appears to be measuring what it is supposed to measure. One method of assessing face validity is to administer the measure to participants and professionals for subjective opinion. Evidence can also be obtained from how the measure was initially developed.

• *Criterion validity -* considers whether scores from the questionnaire correlate with the definitive standard measurement of the same outcome. However, there is no definitive standard for measuring satisfaction in any previous study. A possible exception may be when a longer version of a questionnaire is used as the ‘gold standard’ to develop a shorter version of the same established instrument [22,23]. When no criterion is available it is possible to examine construct validity.

• *Construct validity –* can be used when some attribute (i.e. satisfaction) is not "operationally defined." There are many different methods to assess construct validity:

• *Group differences* - if the understanding of a construct leads to the assumption that groups could differ on the test, this expectation may be tested directly. For example it might be expected that women who experienced low support during birth might have lower patient satisfaction scores.

• *Convergent validity –* if a test has construct validity then it is expected that test scores will correlate with scores on other tests that measure a similar construct.

• *Discriminant validity –* this is the opposite of convergent validity. If different constructs are not considered to be related then there should be no correlation between test scores measuring these different constructs.

• *Factorial validity* – examination of the internal structure of scales and the ability of the construct to provide a clear factor structure.

No statistaical analysis was planned.

## Results

**Figure 1:**
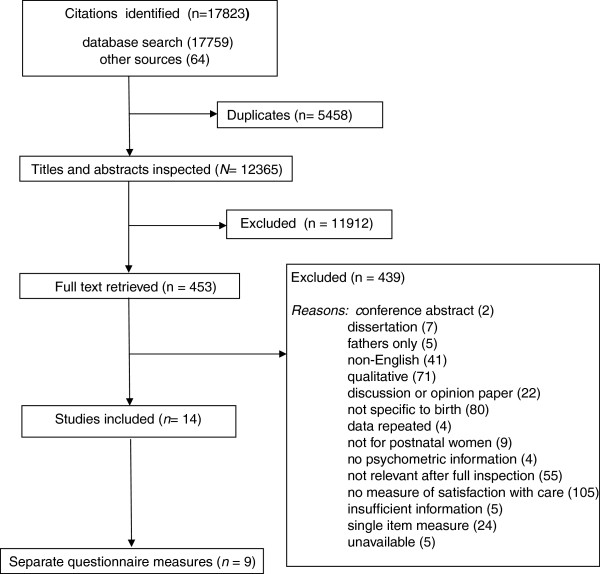
Flow chart.

In total 17,823 citations were identified in the search strategy (Figure 1). After de-duplication and screening of titles and abstracts full text copies were retrieved for 453 citations, of which 439 were excluded. The 14 included studies reported nine measures of satisfaction with care during labour and birth. These studies and the questionnaires are described in Additional file 1 Table S1. For seven questionnaires detail about how the items were selected was reported, and for six a pilot study of the questionnaire was described. Most of the questionnaires (8 out of 9) had tests of internal consistency, but only one reported test-retest reliability [15]. All studies reported on at least one aspect of validity. No study reported criterion validity of the measure. The following section provides a description of each questionnaire and a summary of its psychometric properties.

### Six Simple Questions (SSQ)

The SSQ [5] is a brief, easily administered questionnaire of satisfaction with care during childbirth. The questionnaire has high reliability (α = 0.86). Face validity and content validity is suggested by the questionnaire development, as items were reviewed by the authors who developed the questionnaire and assessed it for congruence with the literature. Also, the questionnaire was administered initially to a small group of women to check clarity of language and ease of administration. Construct validity is suggested by moderate to strong correlation (*r* = 0.51) with another measure, the Labour and Delivery Satisfaction Index (LADSI) [15] (see below), and the finding that women were more satisfied with their care if it was provided by midwives rather than by doctors. The authors used the SSQ to measure satisfaction with childbirth at 48 hours, 2 weeks and 6 weeks postpartum. However, they do not report the correlations between these time points, which would have provided a useful indicator of test-retest reliability.

The SSQ has high reliability and there is evidence of reasonable face validity and content validity; as well as good construct validity. A limitation is that it uses an overall single score for satisfaction, so satisfaction with specific aspects of care cannot be explored.

### Consumer Satisfaction Questionnaire (CSQ)

The CSQ [24] is a 17-item questionnaire developed to measure couples’ perceptions of care during labour and birth. Content and face validity is suggested by the questionnaire development because items were developed from a review of the literature, parent interviews and a pre-test with 20 couples. Factorial validity was established through principal components analysis, which identified three clear factors of satisfaction: supply of equipment; participants in the labour and birth; management of the ward. Together these factors accounted for 65% of the variance. Construct validity is suggested as satisfaction with healthcare was positively associated with social support (*r* = 0.36), especially with medical staff support. Internal consistency for the questionnaire was high (α = 0.93).

The CSQ appears to be reliable and have good face, content and construct validity. However, it has significant limitations. It was developed and validated in Taiwan and, although it is reported in English, has not been validated outside of Taiwan. In addition, the CSQ was validated with couples who had a healthy baby born at 37+ weeks gestation with an uncomplicated vaginal birth. This means it may not be applicable to more complicated births, or births in Western cultures.

### Labour and Delivery Satisfaction Index (LADSI)

The LADSI [15] is a 38-item questionnaire which measures the “technical” and “caring” components of satisfaction. The sample is not described in detail but the authors suggest the measure can be used to assess satisfaction following different types of birth. Content and face validity were established through item generation, as items were created on the basis of a literature review, interviews with women who had recently given birth, and the clinical opinion of investigators. However, the questionnaire was not pilot-tested. Construct validity was established by embedding three mood questions in the tool, and comparing satisfaction between women with high and low mood scores. As predicted, those in the low mood group were less satisfied with care in comparison to those in the high mood group (*p* < .05). Construct validity is also supported by findings from two other studies. One used the LADSI to compare satisfaction with midwifery or doctor-led care [5]. As predicted, women were most satisfied with midwifery-led care. The other used a modified version of the LADSI, [25] and found that satisfaction scores were positively associated with health of mother and baby, perceived control and expectations. Complications with labour and birth were associated with lower satisfaction. Care provided by midwives rather than doctors was also associated with higher satisfaction [25].

The LADSI appears to have face validity and content validity, and there is some evidence of construct validity. Although the questions are phrased so that they can be answered by men, psychometric assessment was not conducted for men. Internal reliability of the measure is questionable. Scores were reasonably stable over the two time points (*r* = 0.64) [15] but Cronbach’s alpha for the questionnaire was low (α = 0.34 overall; caring component α = 0.11; technical component α = 0.78). Moreover, factor analysis did not identify clear factors but yielded only one factor, which explained 28% of the variance, and 10 other factors which did not clearly fit with the caring or technical components. Considering the poor internal reliability and factorial validity the authors suggest only the total score of the questionnaire should be used.

### Maternal Satisfaction for Caesarean Section (MSCS)

The MSCS [26] is a 22-item questionnaire designed to assess womens’ satisfaction with caesarean section under regional anaesthesia. Face and content validity was established through the development of the questionnaire. Women generated items before and after caesarean section and the authors interviewed women until they no longer generated any new suggestions or items for the scale. Existing items from the literature were also included. Construct validity is suggested by a moderate correlation (*r* = 0.48) between the MSCS and a single item measuring overall satisfaction. Additional evidence for the construct validity of the measure is provided by two further studies [27,28]. In the first, the MSCS successfully distinguished between women who had a spinal or epidural anaesthesia for caesarean section, with higher satisfaction scores in women who had an epidural. In comparison, the single satisfaction item was not able to differentiate between the two groups [27]. In the second study higher preoperative anxiety was associated with lower satisfaction in women having elective caesarean section (trait anxiety *r* = -0.24; state anxiety *r* = -0.28) [28]. Factorial validity was established through factor analysis, which produced four clear dimensions: 1) Interaction with Family/Staff; 2) Anaesthetic/Technical effects; 3) Intra/postoperative events; and 4) Side effects. Internal reliability for the total scale and Factors 1 and 2 was high. However, Factors 3 and 4 displayed low reliability. Therefore although the questionnaire appears to have good face, content, and construct validity the reliability is questionable.

### Perceptions of Care Adjective Checklist (PCACL-R)

The PCACL-R [29,30] was originally used in the Great Expectations study [29] which included 15-adjectives (eight negative and seven positive) for women to describe their care during labour and birth. The original paper does not include details of the selection of items or validation of the instrument but a later study identified three factors: negative, supportive, and considerate, which explained 41% of the variance [31]. Redshaw and Martin [30] modified the instrument by including a 16^th^ positive adjective (kind). They administered the questionnaire to a large sample of women who had recently given birth so the instrument could be formally validated. In-depth psychometric analyses were conducted for the checklist. Factorial validity was established through confirmatory factor analysis of the PCACL-R, which revealed a clear two dimensional (positive and negative) structure that fits the data well. The measure also displayed some construct validity. As predicted the PCACL-R was associated with more satisfaction, with the positive checklist related to higher satisfaction, and the negative checklist to dissatisfaction. Divergent validity was also suggested as the checklists, as expected, were not correlated with length of labour. Findings regarding group differences were mixed. Significant discriminability was observed for the positive lists between deprivation and partner status, but not for the negative checklist. This finding highlights the importance of looking at both subscales individually. Contrary to expectations, women who had a non-instrumental delivery scored lower on the PCACL-R positive scale. Both scales also demonstrated predictive validity as they were able to successfully predict satisfaction with communication from healthcare professionals [30]. 

As it is not reported how the items were selected and who reviewed the measure before administration it is difficult to assess content and face validity. However, the survey that provided the data on which the PACL-R was validated was developed on the basis of cognitive interviews and piloted with 400 women, both supporting the face and content validity of the survey instrument which included the PACL-R [32]. Women also reported that it was beneficial that they could report both positive and negative aspects of their care. Reliability was high (total scale α = 0.81; positive scale α = 0.78; negative scale α = 0.73). In summary, the measure has good construct validity and high reliability, as well as some evidence of face and content validity.

### Intrapartal care in relation to WHO recommendations (IC-WHO)

The IC-WHO [33] is an adaptation of a previous questionnaire used to assess quality of maternity care based on the WHO’s recommendations [34]. As such, in comparison to the other questionnaires reviewed in this paper, the focus is on women’s perceptions of the safety of practice and care. Adaptations included revisions to make it understandable for women and the exclusion of questions identified as difficult for women to understand. The questionnaire was tested for legibility and comprehensibility by five women who had recently given birth. The questionnaire includes 63 items that reflect the WHO’s recommendations for care in normal birth and were divided into 1) practices which are demonstrably good and should be encouraged (39 items), 2) practices that are classified as clearly harmful or ineffective (four items), 3) practices where insufficient evidence exists to support a clear recommendation (three items), and 4) practices frequently used inappropriately (16 items). Women were asked to evaluate the items in two ways: the perceived reality (PR) of the care received and the subjective importance (SI) of each item. PR was scored in response to the question “This is what it was like for me” with possible responses being Yes, No, Do Not Know, Not Applicable (scores were finally dichotomised into Yes and No/Do Not Know. SI was measured in response to the phrase “It was this important for me” which was scored on a five-point Likert scale ranging from (1) not important at all to (5) of very great importance. Differences in SI were analysed for each of the 63 items. Although the questionnaire was based on a previous audit instrument which showed acceptable reliability and validity, very little psychometric information for the current measure is reported in the paper. Therefore it is important that this version of the questionnaire is validated for completion by women themselves. As this questionnaire is associated with a normal birth process women who had an elective caesarean section were excluded, which means it may not be suitable for this sample of women.

### Patient Perception Score (PPS)

The PPS [35] is a brief, easily administered questionnaire of satisfaction with care during childbirth. The measure consists of three-items that assess satisfaction with communication, safety and respect. The questionnaire has previously been used in trials of Simulation and Fire Drills Evaluation, including simulated obstetric emergencies. Validation of the PPS was done on 150 women following an operative birth. Face and content validity is suggested as the measure was approved by a focus group, funding body and ethics committee. However, although the three dimensions assessed are identified in the literature as important components of satisfaction during childbirth, the PPS is a very short and simple questionnaire and content validity cannot be assured. Construct validity was established in a number of different ways. Firstly, the PPS was strongly (r = 0.64) correlated with a modified version (inclusion of only medical care items) of the Mackey Childbirth Satisfaction Rating Scale (MCSRS) [1]. Secondly, the measure was able to distinguish between seniority of staff, with lower levels of staff receiving lower ratings of satisfaction. The three items also showed high reliability (α = 0.83). The administration of the PPS also appears feasible as women reported the PPS to be simple and easy to complete. In summary the measure shows good construct validity and has high reliability, but due to the brevity of the measure face and content validity cannot be guaranteed.

### Questionnaire to assess clients’ satisfaction (CliSQ)

The CliSQ [36] is a 39-item questionnaire which measures three aspects of satisfaction: environment condition, care procedures and provided education. Total scores are converted into percentages and bands of 0–39, 40–59 and 60–100 are used to represent dissatisfaction, neutral, and satisfaction respectively. Psychometric validation of the measure was fairly limited. The questionnaire was not pilot tested before use and it is not clear how the items were generated. Attempts at content and face validity were made as the checklist was reviewed by 15 midwives and obstetricians. There is some evidence of the construct validity of the measure as there was a small correlation (*r* = 0.34) between women’s desired care and their satisfaction with care. Factorial validity was not established, despite the authors reporting three subscales of satisfaction with care. However, the total scale showed high reliability (α = 0.83).

### Intrapartal-Specific QPP-questionnaire (QPP-I)

The QPP-I [16] was developed from a general measure of satisfaction: the Quality from the Patient’s Perspective questionnaire (QPP) [37] which has a theoretical foundation. Twenty-two items of the QPP-I are based on items from the short [38] and long version of the QPP [39]. Ten items were newly constructed, which were derived from the IC-WHO measure based on WHO recommendations [33,34]. Items were informed by previous questionnaires and interviews with women suggesting the measure has content and face validity. Women were asked to evaluate the items in two ways: the perceived reality of the care received and the subjective importance of each item. Factorial validity was established through structural equation modelling, which revealed one general factor and 10 subordinate factors. The measure also displayed some construct validity as women who scored higher on the perceived reality items were more likely to return to the same ward in the future. Reliability was mixed (perceived reality subscales α range = 0.50 to 0.92; subjective importance subscales α range = 0.49 to 0.93). The subscales with poor reliability were ‘midwives present during labour’ and ‘medical care and pain relief’ with alphas of 0.50 and 0.58 respectively (perceived reality), and ‘midwives present during labour’ and ‘participation’ with alphas of 0.49 and 0.57 respectively (subjective importance). Some of the factors only have a few items, which could contribute to reduced reliability [40]. The QPP-I has therefore good face, content, and construct validity; but mixed reliability.

## Discussion

The purpose of this review was to identify questionnaires that measure satisfaction with care during labour and birth, and to examine their psychometric quality. Nine questionnaires were identified and evaluated. Questionnaires varied in the extent and quality of psychometric evaluation, as well as in their application to different populations (e.g. normal versus caesarean birth). Results from this review can help inform the decision about which questionnaire to use for particular circumstances.

### Choosing the appropriate questionnaire

Most of the questionnaires were intended to measure satisfaction with care during all types of labour and birth. Of these, the LADSI is probably the most frequently used but has low reliability and an unclear factor structure. The CliSQ showed adequate internal consistency but there is no information on how the items were generated, and items were only reviewed by healthcare professionals to assess content and face validity. The QPP-I and the IC-WHO were the only questionnaires that assessed satisfaction with different aspects of care as well as the perceived importance of these aspects of care. Indeed, a major limitation of satisfaction studies is that the relative importance or values that individual women or their partners place on different aspects of childbirth has seldom been measured [11]. Of these two measures, the IC-WHO is lengthy and no reliability or validity statistics were reported. In comparison the QPP-I is shorter, based on a theoretical model of patient satisfaction, was developed partly on the basis of interviews with women, and shows reasonable reliability and strong validity. Therefore, if an in-depth measure of satisfaction with maternity services is required the QPP-I would be most appropriate.

For quick, simple assessment the SSQ or PCACL-R are probably most appropriate, with both demonstrating good reliability and validity overall. An advantage of both these measures is that they can be used to evaluate other aspects of maternity care (antenatal and postnatal) if needed. However, whilst the PCACL-R asks participants to describe their care using positive and negative adjectives, it does not explore individual aspects of care (such as information provision, and involvement in decision making) so is less useful for evaluating particular aspects of maternity care.

Finally, three questionnaires were designed for particular types of births. Two questionnaires were designed for operative births (PPS and MSCS) and one for uncomplicated vaginal birth (CSQ). For operative births the PPS provides a brief, three-item measure which can be easily administered and completed quickly in a clinical setting. It has also been validated with women who have caesarean section or assisted birth (ventouse/forceps). The MSCS is a more detailed questionnaire with four clearly defined factors and good validity [27,28]. However, it was only validated in women who had elective caesarean sections, so may not be appropriate for women who have emergency caesareans. The only measure validated with women who had uncomplicated, vaginal birth was the CSQ. This measure has very good psychometric properties and can be used with mothers and fathers. However, the questionnaire has only been validated in Taiwan so requires further validation if used in other countries.

Finally, it should be noted that although some questionnaires were designed for specific birth types, no questionnaires were identified that evaluated care in specific populations, such as parents of sick or preterm babies, and parents who baby was stillborn. The experience of parents during these types of birth may be substantially different from giving birth to a healthy, term baby. Therefore, current measures of satisfaction with care may not be suitable for such parents. For example, a qualitative study exploring parents’ views of care during the birth of their very premature baby found that staff appearing calm during the birth was an important contributor to satisfaction with care, a domain which is not included in current measures of birth satisfaction [41].

### Issues with measuring satisfaction with maternity care

This review has highlighted a number of issues surrounding the assessment of satisfaction. Firstly, there are only a small number of studies which include a multi-item measure of satisfaction with care specifically during labour and delivery. Amongst those that do, very few also include some form of psychometric detail/evaluation. However, the search did identify a large number of studies which assessed satisfaction with maternity care services at different time points or in general and were therefore not included in this review [13,42,43]. These types of omnibus measures are not as useful for healthcare providers or researchers who need a simple, quick measure specifically for care in labour and delivery. However, inspection of the items used in these studies would be helpful to authors who are constructing a childbirth satisfaction measure.

Secondly, the studies in this review varied greatly in when they measured satisfaction (ranging from 24 hours to 15 weeks after birth), and some studies did not include any information about when satisfaction was measured. There are indications that the time of administration of a survey to measure satisfaction has an influence on satisfaction ratings. For example, satisfaction with care can change even over a short time [44] and measurement of satisfaction whilst the mother is still in hospital could produce different ratings compared to after discharge. Assessment of satisfaction with childbirth may be more suited when a certain time lag has passed following birth, as this will give the mother time to reflect on her experience and decide whether she was satisfied. Waldenstrom [45] examined why some women’s opinions became more negative a year after birth and found that soon after the birth women’s responses may be overshadowed by relief that labour was over and the birth of a healthy baby. Longer after the event women may be more prepared to face negative aspects of labour and birth, such as having a long labour, medical interventions, or an unsatisfactory relationship with the caregiver.

Thirdly, although it is important to recognise the association between a woman’s experience of labour and birth (such as pain and negative emotional experience) and her evaluation of care, studies do not always differentiate between the two [17,19]. For example, a large number of questionnaire measures were excluded that included items that assessed both satisfaction with the experience and satisfaction with care [1,46,47]. Although satisfaction is a complex construct that can be difficult to define, it is important that authors clearly outline the definition and conceptualisation of patient satisfaction used in their studies.

Fourthly, in a review of patient satisfaction measures, a theoretical foundation was highlighted as an essential criterion for satisfaction measures [48]. The only measure which explicitly stated that it was developed on the basis of a theoretical model of patient satisfaction was the QPP-I. Most of the measures were data driven and were not based on the main theoretical models of satisfaction. For example, none of the measures incorporated expectation fulfilment into their assessment, which has been shown to be particularly important for women during childbirth [1,3].

Finally, the strict inclusion and exclusion criteria should be considered when evaluating the results of this review. Forty one papers were excluded because they were not published in English, meaning that additional measures may have been identified if these were included. In addition measures were excluded if any of the items were not about the birth i.e. included antenatal and/or postnatal items, or were not specifically concerned with care, which again may have resulted in a larger number of measures.

### Issues surrounding validation of the questionnaires

Exploring the factor structure of a questionnaire is viewed as an essential part of questionnaire design [49] as it not only provides a basis for removing redundant or unnecessary items but can identify the underlying domains or subscales of a questionnaire. Some measures (e.g. CliSQ, IC-WHO) reported a multi-dimensional measure of childbirth satisfaction but the internal structure was not validated by factor analysis. Therefore the factorial validity of these measures is questionable and should be addressed by future research. Likewise, two questionnaires (SSQ, PPS) had very few items which means the factor structure cannot be explored.

Another important issue in validating questionnaires is ensuring this is done in an appropriate population with adequate sample size to be representative of that population. All of the questionnaires were validated in the appropriate populations (i.e. postnatal women, normal, instrumental, and caesarean deliveries). However, with the exception of the PCACL-R and QPP-I, most studies consisted of relatively small sample sizes. Finally, all but two measures were designed and phrased specifically in relation to childbirth, which may increase the validity of these measures. However, the two generic questionnaires (SSQ and PCACL-R) may be particularly useful to compare satisfaction at different time points.

## Conclusions

Despite the plethora of research examining satisfaction with maternity care, only a small number of validated measures have looked specifically at satisfaction with care during labour and birth. Satisfaction is an increasingly reported outcome which requires brief, reliable and valid measures for use generally and with specific populations to inform practice. This review provides an overview of the current measures and recommends questionnaires for the particular needs of the researcher and/or clinician. Future research should continue to evaluate and report psychometric properties of these measures. This information will enable clearer choices regarding valid and reliable measures. In addition, research needs to consider timing of measurement, as well as being clear about how satisfaction is conceptualised and the theoretical basis for this.

## Competing interests

The authors declare that they have no competing interests.

## Authors’ contributions

AS contributed to the protocol, carried out the searches, analysed the results and drafted the manuscript; SA contributed to the protocol, analysis and revisions of the manuscript; JA and GG (representatives from parent groups) contributed to the protocol and revisions of the manuscript; HR and LD contributed to the protocol and revision of the manuscript. All authors read and approved the final manuscript.

## Pre-publication history

The pre-publication history for this paper can be accessed here:

http://www.biomedcentral.com/1471-2393/13/108/prepub

## Supplementary Material

Additional file 1: Table S1 Characteristics of included questionnaires. Click here for file
