# Supplementary material for: Measures of satisfaction with care during labour and birth: a comparative review
Source: BMC Pregnancy Childbirth. 2013 May 8;13:108. doi: 10.1186/1471-2393-13-108 (PMC3659073; doi:10.1186/1471-2393-13-108)
Supplement: Additional file 1: Table S1 — Characteristics of included questionnaires. [file 1471-2393-13-108-S1.docx]

**Table S1. Characteristics of included questionnaires**

| **Questionnaire** | **Format** | **Dimensions** | **Country** | **Sample** | **Questionnaire Construction** | **Reliability** | **Validity** |
| --- | --- | --- | --- | --- | --- | --- | --- |
| **Six Simple Questions (SSQ) ^[5]^** | 6 items using a 7-point Likert Scale.  Possible scores range from 7 to 42. | NR | Canada | 194 women  48 hours postpartum, 2 weeks postpartum and 6 weeks postpartum | *Item generation:* items selected by investigators, review of literature  *Pilot study:* small convenience sample | *Internal consistency* **=** 0.86  *Test re-test reliability* = NR | *Face* **–** items selected by investigators, review of literature and then administered to a group of women  *Content*– assessed by members of research team, inspection of the literature, and a small group of women  *Criterion* - NR  *Construct (group differences)* – assessed differences in satisfaction with care provided either by midwives or doctors  *Construct (convergent)* – SSQ was associated with LADSI |
| **Consumer Satisfaction Questionnaire (CSQ) ^[24]^** | 17 items using a 5-point Likert scale. Possible scores range from 17-85. | 1) Supply of equipment,  2) Participants in the birth,  3) Management of the ward | China | 114 wives and 77 husbands (between 48-72hrs after birth)  Uncomplicated, vaginal delivery | *Item Generation –* review of literature, patient interviews, pre-test study.  *Pilot study* – 20 couples completed the questionnaire | *Internal consistency* **=** 0.93  *Test re-test reliability* = NR | *Face –* items were developed from a review of the literature and patient interviews.  *Content –* items were developed from a review of the literature and patient interviews.  *Criterion* - NR  *Construct (factorial) –* PCA identified a clear 3-factor structure  *Construct (group differences)* – explored correlations between social support and satisfaction. |
| **Labour and Delivery Satisfaction Index (LADSI) ^[15] [5] [25]^** | 38 items using a 6-point Likert scale. Possible scores range from 38-228. | 1) Technical component,  2) Caring component | Canada | 59 women (2 days postnatal)    35 women (4-6 weeks after birth) | *Item Generation* – review of literature, patient interviews, clinical opinion of investigators  *Pilot* - NR | *Internal consistency*:  Total = 0.35  Caring subscale = 0.11  Technical subscale = 0.78    *Test-retest* = 0.67 | *Face* – items reviewed by obstetricians and nurses for wording and face validity.  *Content –* items developed through review of literature, patient interviews, and clinical opinion of investigators  *Criterion* - NR  *Construct (group differences)* – correlations explored between mood scores and satisfaction  *Construct (factorial)* – factor analysis produced 1 general factor and 10 unclear factors. |
| **Maternal Satisfaction for Caesarean Section (MSCS) ^[26] [27] [28]^** | 22 items (7-point Likert scale).  Possible scores range from 22 to 154. | 1)Interaction with family/staff, 2)Anaesthetic/technical effects, 3)Intral/postoperative effects, 4) Side effects | Canada | 115 women who had a caesarean section | *Item Generation* **–** review of items used in previous studies, interviews with mothers.  *Pilot* - NR | *Internal consistency*:  Total = 0.82  Interaction with family/staff) = 0.69  anaesthetic/technical effects = 0.70  intral/postoperative effects = 0.45  side effects = 0.45  *Test-retest* = NR | *Face* – women generated items before and after caesarean section  *Content* – Interviewed 25 women until no new items were suggested, review of literature  *Criterion* - NR  *Construct (convergent)* – explored correlation between scale and VAS satisfaction item.  *Construct (factorial)* – PCA identified four clear factors |
| **Perceptions of Care Adjective Checklist Revised (PCACL-R) ^[30]^** | 15 items in original version and 16 in adapted version. | 1) Positive adjectives,  2) Negative adjectives | UK | 2960 women  (*M* = 15.5 weeks since birth) | *Item generation* - NR  *Pilot* – NR | *Internal consistency:*  Total = 0.81  Positive = 0.78  Negative = 0.73  *Test-retest* = NR | *Face –* NR  *Content –* NR  *Criterion -* NR  *Construct (group differences)* – relation between satisfaction and staff communication, SES, marital status, type of delivery  *Construct (convergent)* – relationship between scale and a single item of satisfaction.  *Construct (divergent)* – relationship between labour length and satisfaction.  *Construct (factorial) –* CFA identified a two factor correlated model. |
| **Women’s perception of intrapartal care in relation to WHO recommendations**  **(IC-WHO) ^[32]^** | 63 items  Perceived reality and subjective importance assessed. | 1) Practices which are good and should be encouraged,  2) Clearly harmful or ineffective practices,  3) Insufficient evidence to support recommendation,  4) Practiced frequently and used inappropriately | Sweden | 140 women (approx. 2 months postpartum) | *Pilot:* 5 women who had recently given birth | *Internal consistency* **=** NR  *Test retest reliability* = NR | *Face –* questionnaire initially administered to 5 women for feedback  *Content -* questionnaire initially administered to 5 women for feedback  *Criterion* - NR  *Construct* - NR |
| **Patient Perception Score (PPS) ^[34] [40]^** | 3 items using a 5-point Likert Scale. Possible scores range from 3-15. | 1) Communication  2) Respect,  3) Safety | UK | 150 women  Operative delivery (Caesarean, Ventouse, Forceps)  Completed questionnaire within 24 hours after birth | *Item Generation* - used a questionnaire used in previous trials of “Simulations and Fire Drills Evaluation” including obstetric emergencies.  *Pilot study* - current study is reported as a pilot study. | *Internal consistency* = 0.83  *Test re-test reliability* = NR | *Face* – approved by focus group, ethics committee, funding body  *Content*– approved by focus group, ethics committee, funding body  *Criterion* – NR  *Construct (group differences) -* compared satisfaction with staff of differing levels of experience.  *Construct (convergent)* – explored correlations between PPS and Mackey’s CSRS. |
| **Client Satisfaction Questionnaire (CliSQ) ^[35]^** | 39-item scale using a 5-point Likert Scale. Possible scores range from 39 to 195. | Environmental condition, Care procedures, Provided education | Iran | 96 women (gestation 37 to 41 weeks). | *Item generation* - NR  *Pilot* – NR | *Internal consistency* = 0.7  *Test-retest* = NR | *Face -* reviewed by midwives and obstetricians  *Content* – reviewed by midwives and obstetricians  *Criterion* - NR  *Construct* (group differences) – explored correlation between satisfaction and compatibility with desired care. |
| **Intrapartal-Specific QPP-Questionnaire (QPP-I) ^[16] [40]^** | 32 items measuring perceived reality and subjective importance were assessed using a 4-point Likert scale. | 1) Medical care and pain relief,  2) care equipment and care room,  3) information before procedures, 4) information on self-care,  5) participation,  6) commitment, empathy, respect (doctors),  7) commitment, empathy, respect (midwives),  8) commitment, empathy, respect (nurses),  9) midwife present during labour,  10) partner/ significant other. | Sweden | 739 women (approximately 2 months postpartum) | *Item development:* 22 items selected from short and long versions of the QPP and 10 new items were newly constructed  *Pilot:* 20 women completed the questionnaire | *Internal consistency (*PR and SI*)* **:** 1)Medical care and pain relief (0.53 and 0.73), 2) care equipment and care room (0.73 and 0.81), 3) information before procedures (0.77 and 0.79), 4) information on self-care (0.80 and 0.86), 5) participation (0.61 and 0.57), 6) commitment, empathy, respect (doctors) (0.92 and 0.93), 7) commitment, empathy, respect (midwives) (0.73 and 0.70) 8) commitment, empathy, respect (nurses) (0.90 and 0.92), 9) midwife present during labour (0.50 and 0.49), 10) partner/significant other (0.76 and 0.85)  *Test re-test reliability* = NR | *Face -* QPP items inspired by women’s interviews, new items based on previous questionnaire and were reviewed by women  *Content* **–** QPP items inspired by women’s interviews, new items based on previous questionnaire and were reviewed by women  *Criterion* - NR  *Construct (group differences)* - Women who scored higher on the perceived reality items were more likely to return to the same ward in the future  *Construct (factor)* – SEM identified one general factor and 10 sub-factors. |

Note. NR = Not Reported, PCA = Principal Components Analysis
